# Notes and News

**Published:** 1902-10-11

**Authors:** 


### Notes and News.

Princess Beatrice visited the Boscombe Hospital on Monday last, and expressed herself much pleased with the institution.

Lord Llangattock has laid the foundation-stone for a new hospital for Monmouth, to which he has subscribed £2,000. The total cost is estimated at £7,500.

Mr. Alfred Willett has been entertained at a dinner given by his old house-surgeons on his retirement from his post of surgeon at St. Bartholomew's Hospital. At the same time he was presented with a portrait-bust of himself.

The Metropolitan Asylums Board is asking the Local Government for permission to classify the patients they receive and to create two classes. The proposal is aimed at separating the less desirable patients from the more respectable classes, which can be effected by the introduction of payments, which will give the patients the option of entering under the classification which is most suitable.

The new Dundee Sanatorium for Consumptives at Auchterhouse stands 800 feet above the level of the sea. The bedrooms all face south, and below is a fine terrace. The windows are large, but the bedrooms do not open on to any verandah or balcony. There is accommodation for about forty patients in the main block. Lord Airlie presented the site, and ex-Provost Moncur contributed £10,000 and later on another £5,000 to the scheme.

The foundation-stone of a new sanatorium for consumption has been laid at Delamere. The institution is being built, through the munificence of Mr. W. J. Crossley, for the use of the Manchester and Salford Hospital for Consumption and Diseases of the Throat. The cost of the sanatorium will be £70,000. It has a site of 70 acres, and will stand 450 feet above the level of the sea. Ninety patients are to be accommodated, and a detached home will be provided for the nursing and domestic staff. There will be four wards for six beds, ten for four beds, and the remainder will be single rooms. There will be sitting-rooms and balconies. The windows throughout will open to the floor level. The architect is Mr. W. Cecil Hardesty, of Manchester.

There is still a considerable amount of cholera in Egypt.

Professor Johannes Orth, of Gottingen, will succeed Professor Virchow as Professor of Pathology in Berlin. He acted as Virchow's assistant at Berlin for some time.

Mr. Fowke, the general secretary of the British Medical Association, has retired from the post he has occupied since 1871, owing to ill-health. His services will be much missed. He has been appointed an honorary member of the association in recognition of his valuable work. He has been succeeded by Mr. Guy Elliston.

Although there have been no cases of plague recently in Sydney, precautions are not being relaxed. About 800 rats have been examined at the public health laboratories each week, and an order has been issued to the owners of all premises in Sydney having gratings or areas such as would admit rats, to cover them with a fine wire-netting so as to effectually prevent their entry.

The foundation-stone of the new isolation hospital at Penarth has been laid by Mr. S. Thomas, chairman of the District Council. The hospital will stand on a site of three and a half acres, and will consist of an isolation block, with two wards and two beds in each; a fever block, with two wards for six beds in each; an administration block, and laundry block, in which will be the disinfecting apparatus and mortuary. The estimated cost is £7,800, including the furnishing, and the architects are Mr. E. Evans and Mr. H. Cornish.

The affairs of the Manchester Royal Infirmary have reached a curious crisis. The Board of Management wish to rebuild on the present site, but the trustees are definitely opposed to any such proceeding. It cannot be doubted that the general feeling of the Manchester public is with the majority of the trustees in favour of removing the Infirmary from Piccadilly to a quieter situation away from the centre of the city, and nearer than the present site is to the Owens College, where the medical school has its home. Under these circumstances, and in view of the absolute divergence of opinion between the two bodies who control the affairs of the Infirmary, the Board of Management have decided to resign as soon as their successors can be appointed. Thus the whole matter is thrown into the melting pot once again, and the appeal will be to the general mass of subscribers who will practically be asked whether they will support the Board, the retention of the old site, and the limitation of the progress of the hospital in deference to the old sentiment in favour of keeping the hospital in Piccadilly, or will start afresh with all the advantages of having a free hand, an open site, a purer air, and a more convenient situation in regard to the medical school. As to the old argument of Piccadilly being central, we cannot but think that Manchester is far too great a city, and the district served by the Manchester Royal Infirmary covers far too large an area, for the difference of a mile or so between the sites in Piccadilly and Stanley Grove to be a matter of any real importance.

